# Effects of external diaphragmatic nerve stimulation on diaphragm and cortical activity in stroke patients: a functional near-infrared spectroscopy study

**DOI:** 10.3389/fneur.2026.1786178

**Published:** 2026-04-10

**Authors:** Xiu-nan Huang, Ya-lin Huang, Pei-shu Li, Yi-en Meng, Jin Xu, Chong-wu Xiao, Yao-bin Long

**Affiliations:** 1 The Second Clinical Medical College of Guangxi Medical University, Nanning, Guangxi, China; 2 Department of Rehabilitation Medicine, The Second Affiliated Hospital of Guangxi Medical University, Nanning, Guangxi, China; 3 School of Oncology, Guangxi Medical University, Nanning, Guangxi, China

**Keywords:** cortical activation, diaphragm, external diaphragmatic nerve stimulation, functional near-infrared spectroscopy, stroke

## Abstract

**Background:**

Diaphragmatic dysfunction following stroke severely impacts patient prognosis. However, the central neuromodulatory mechanisms of External Diaphragmatic Nerve Stimulation (EDNS) across different stroke subtypes, including ischemic Stroke (IS) and Intracerebral Hemorrhage (ICH), remain unclear.

**Objective:**

To investigate the immediate effects of EDNS on peripheral Diaphragm Excursion (DE) and central cortical activation in stroke patients, and to compare the response heterogeneity between IS and ICH subtypes.

**Methods:**

This prospective observational study included 50 convalescent stroke patients (30 IS and 20 ICH) and 15 age- and sex-matched healthy Controls (HC). All participants received a single 20-min EDNS intervention. DE was measured using M-mode ultrasound at three time points (pre-, during, and post-intervention). Concurrently, functional Near-Infrared Spectroscopy (fNIRS) was used to synchronize monitoring of cortical activation across 10 Regions of Interest (ROIs). Data were strictly evaluated using non-parametric tests with false discovery rate (FDR) correction.

**Results:**

EDNS intervention significantly improved immediate diaphragmatic function across all groups. DE in the IS group increased from 1.67 ± 0.29 cm at baseline to 1.92 ± 0.33 cm during intervention (*p* < 0.01), and in the ICH group from 1.64 ± 0.26 cm to 1.87 ± 0.31 cm (*p* < 0.01). However, t the spatial pattern of central activation exhibited significant heterogeneity: the HC group showed no significant activation in any ROI (*p* > 0.05); the IS group activated a broad “cognitive-sensory” activation pattern, including the bilateral Dorsolateral Prefrontal Cortex (DLPFC) (*p* < 0.05) and bilateral Somatosensory Association Cortex (SAC) (*p* < 0.01); whereas the ICH group activated only a localized sensory region (Right SAC, *p* = 0.046). Furthermore, the magnitude of DE improvement was significantly positively correlated with the intensity of Right DLPFC activation only in the IS group (Spearman’s *ρ* = 0.386, *p* = 0.035), with no such correlation observed in the ICH group (*p* > 0.05).

**Conclusion:**

Although EDNS universally improves immediate diaphragmatic function in both IS and ICH patients, the underlying central neuromodulatory mechanisms are subtype-specific. IS patients appear to preserve a “top-down” cognitive-sensory regulation pathway (DLPFC-SAC) and central-peripheral coupling, whereas the central response in ICH patients is limited to basic sensory processing (SAC) with a lack of such coupling. This mechanistic disparity suggests that rehabilitation strategies for IS patients might benefit from integrating cognitive engagement, highlighting the necessity of tailoring personalized respiratory rehabilitation protocols based on stroke pathology.

## Introduction

Stroke is a primary cause of long-term disability worldwide (1, 2). While limb dysfunction is widely recognized, respiratory dysfunction secondary to central nervous system injury, particularly Diaphragmatic Dysfunction (DD), is equally common and severe (3). Weakness of this core respiratory muscle not only reduces ventilation efficiency but also severely impairs cough clearance ability, thereby significantly increasing the risk of stroke-associated pneumonia, respiratory failure, and even death (4, 5). Previous studies have confirmed that, compared with healthy individuals, stroke patients generally exhibit reduced diaphragmatic function. This decline is closely associated with limb motor recovery, balance ability, and overall prognosis (6, 7). Since this impairment originates from the interruption of efferent central corticospinal pathways, targeting diaphragmatic function is of great significance in neurorehabilitation.

External Diaphragmatic Nerve Stimulation (EDNS) is a non-invasive technique designed to activate the diaphragm by stimulating the phrenic nerve (8). Its principle is analogous to Functional Electrical Stimulation (FES) used in paralyzed limbs to counteract disuse atrophy (9, 10). Current literature has extensively documented the peripheral efficacy of EDNS. Previous studies utilizing ultrasound imaging have confirmed that EDNS can significantly increase Diaphragm Excursion (DE) and Thickening Fraction (TF) in stroke patients, thereby improving ventilation volume (11, 12). Furthermore, recent trials have suggested that electrical stimulation of the phrenic nerve prevents disuse atrophy of diaphragm muscle fibers and facilitates the recruitment of motor units (13).

However, focusing solely on peripheral muscle mechanics provides an incomplete picture. Respiration is regulated by a bidirectional ‘central-peripheral coupling’ mechanism: cortical drive initiates breathing, while sensory feedback from the diaphragm modulates cortical excitability (14). While the peripheral benefits of EDNS are well-established, its central neural mechanisms remain a critical knowledge gap. Stroke is fundamentally a brain injury, and any lasting functional recovery relies on neural reorganization (15). It remains unclear how this “bottom-up” stimulation via phrenic nerve afferents is processed by the injured brain, and whether it can modulate cortical networks involved in sensorimotor integration and higher-order cognitive control (16, 17). Functional Near-Infrared Spectroscopy (fNIRS) offers a robust, non-invasive method to monitor these cortical hemodynamic responses in real-time, providing a distinct advantage over fMRI by tolerating moderate body movements associated with respiratory interventions (18, 19).

A critical knowledge gap remains regarding the differential responses of stroke subtypes to respiratory neurorehabilitation. Ischemic Stroke (IS) and Intracerebral Hemorrhage (ICH) are characterized by distinct pathological mechanisms, involving focal tissue necrosis in IS compared to the mass effect and hematoma-induced neurotoxicity in ICH (20, 21). These fundamental differences may result in divergent patterns of cortical diaschisis and interhemispheric inhibition. Consequently, it is scientifically plausible that the “central-peripheral coupling” mechanism modulated by EDNS varies significantly between these two subtypes. However, no prior study has utilized simultaneous fNIRS and ultrasound to quantify these potential differences. Therefore, the primary aim of this study was to employ a multimodal approach to: (1) investigate the immediate effects of EDNS on central-peripheral coupling; and (2) characterize the specific differences in neural and muscular responses between IS and ICH patients.

## Methods

### Study design and ethics statement

This study employed a prospective observational design and was conducted in accordance with the Declaration of Helsinki. The study preparation phase was initiated in October 2023. Actual participant recruitment and data collection were conducted from October 2024 to September 2025, strictly following the approval of the Ethics Committee of the Second Affiliated Hospital of Guangxi Medical University (Approval No: 2024-KY1058). Written informed consent was obtained from all participants or their legal guardians prior to enrollment.

### Participants stroke group

All patients were diagnosed and classified according to the latest Chinese guidelines for the diagnosis and treatment of cerebrovascular diseases (22, 23), with IS or ICH confirmed by cranial computed tomography (CT) or magnetic resonance imaging (MRI). Inclusion criteria were: (1) age 35–75 years; (2) in the convalescent stage (3 weeks to 6 months post-onset); (3) stable vital signs, clear consciousness, and ability to follow commands; and (4) absence of severe cardiopulmonary dysfunction (defined as New York Heart Association (NYHA) functional class ≥ III or severe chronic obstructive pulmonary disease). Exclusion criteria were: (1) significant cognitive impairment preventing cooperation with experimental procedures (Mini-Mental State Examination (MMSE) score < 24) or psychiatric disorders; (2) contraindications to EDNS (e.g., skin lesions in the stimulation area, pneumothorax, implanted cardiac pacemakers); (3) presence of other central nervous system diseases; or (4) use of medications affecting neuromuscular excitability.

### Healthy controls (HC)

**Figure 1:**
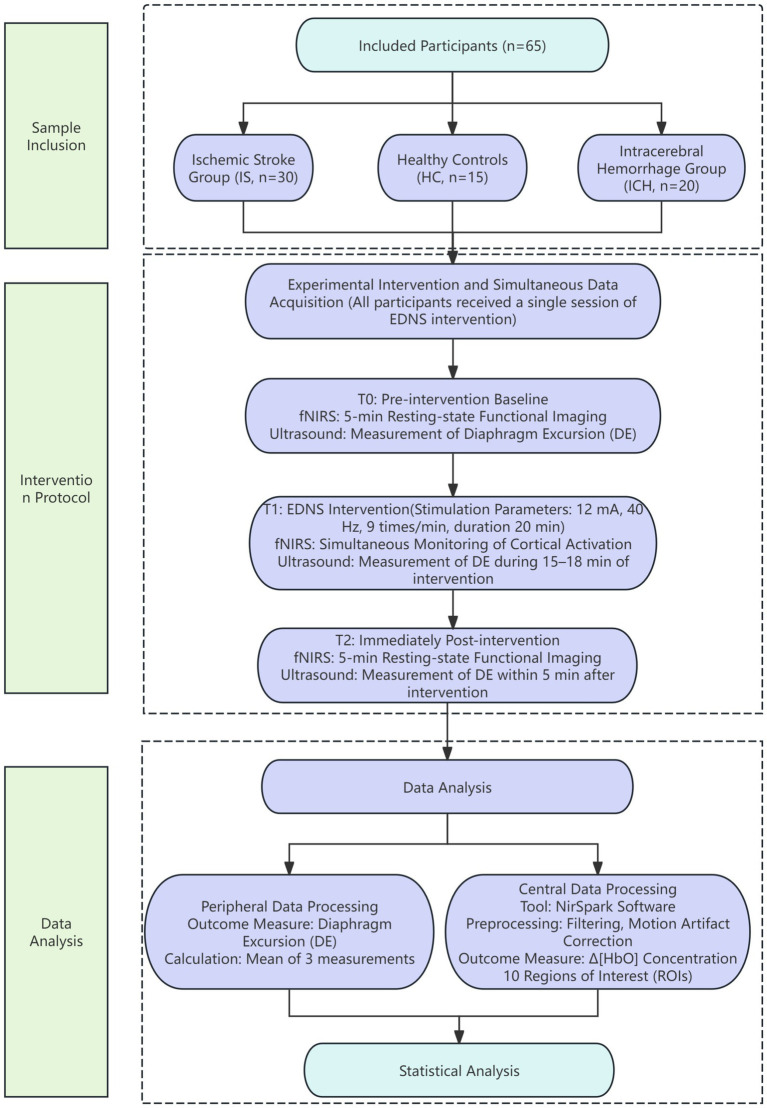
Flowchart of the study design and experimental protocol.

Fifteen age- and sex-matched healthy volunteers were recruited during the same period to establish a baseline for physiological cortical responses. Inclusion criteria were: (1) no history of neurological or psychiatric diseases; (2) no history of severe cardiopulmonary diseases; and (3) normal cognitive function (MMSE score ≥ 28) to rule out undiagnosed mild cognitive impairment and establish a rigorous physiological baseline for cortical activation comparison. The HC group underwent the exact same intervention and data acquisition procedures as the patient group. The inclusion and exclusion criteria for the experimental samples are shown in Figure 1.

### Intervention protocol

**Figure 2:**
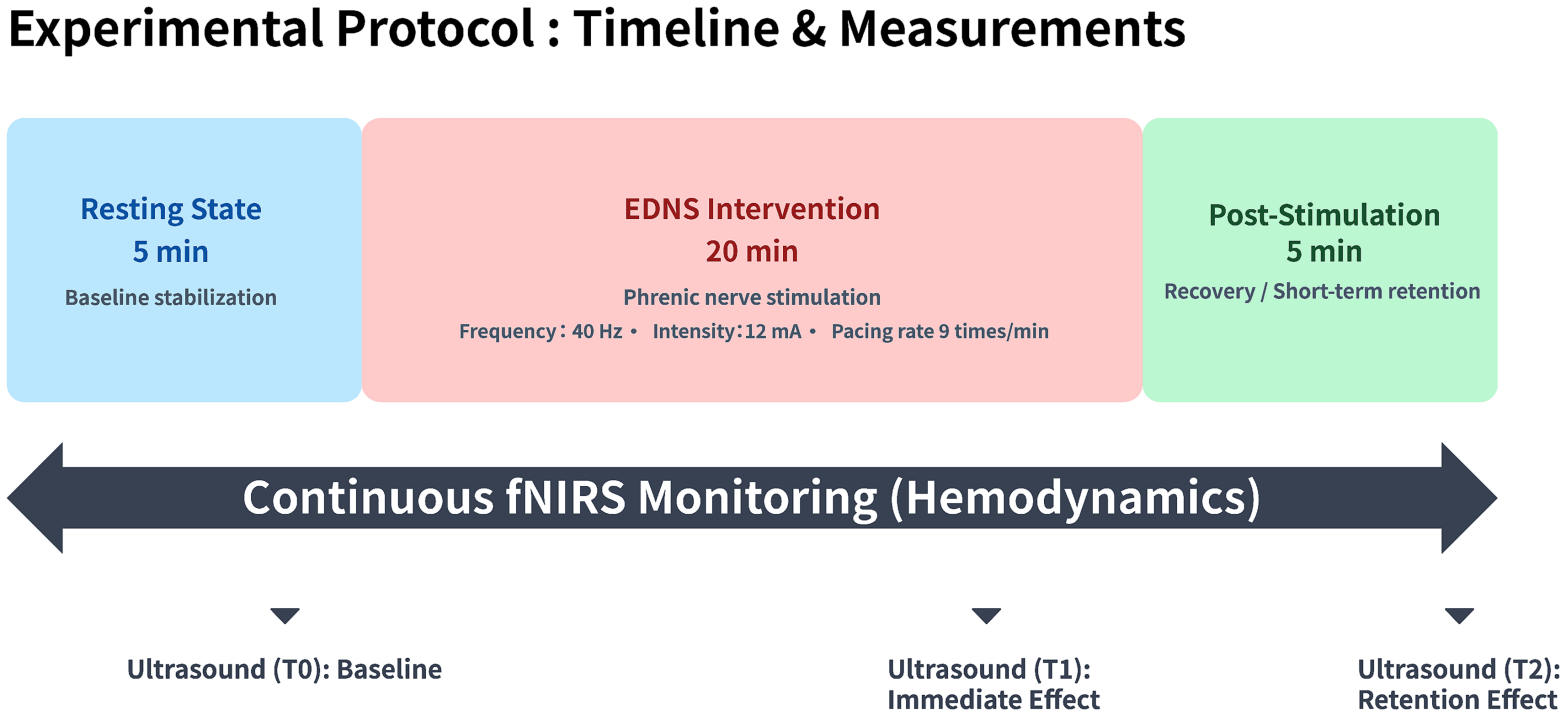
Schematic diagram of the experimental protocol. The study paradigm consisted of three consecutive phases: a 5-min resting state (baseline), a 20-min EDNS intervention, and a 5-min post-stimulation period. Continuous monitoring: Cortical hemodynamic responses were monitored continuously using fNIRS throughout the entire 30-min session. Time points: Diaphragmatic function was assessed using ultrasound at three specific time points: T0 (baseline), T1 (immediate effect during stimulation), and T2 (retention effect post-stimulation).

All participants received a single session of standardized EDNS. A low-frequency extracorporeal diaphragm pacemaker (Model HLO-GJ13A, Guangzhou Shirleyang Biotechnology Co., Ltd., Guangzhou, China) was used with participants in the supine position. Stimulation electrodes were placed at the junction of the lower one-third of the outer edge of the sternocleidomastoid muscle bilaterally (surface projection of the phrenic nerve) (13). Stimulation parameters were set as follows: frequency 40 Hz, pacing rate 9 times/min, with pulse width and waveform set to the device’s standard defaults (24). The current intensity was initially set at 12 mA (or adjusted to the minimum threshold required to induce comfortable, slight neck muscle twitching and rhythmic diaphragmatic contraction) (25). The duration of a single intervention session was 20 min. The detailed experimental timeline and data acquisition protocol are illustrated in Figure 2.

### Outcome measures

#### Diaphragm excursion (DE)

**Figure 3:**
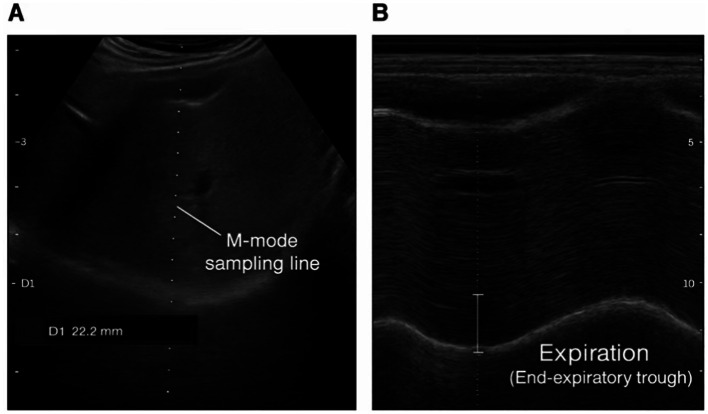
Methodological illustration of ultrasound assessment for diaphragmatic function. **(A)** B-mode anatomical *positioning:* the convex probe was placed subcostally on the right side, using the liver as an acoustic window. The dashed vertical line indicates the M-mode sampling line, positioned perpendicular to the posterior third of the diaphragm. **(B)** *M-mode tracing of diaphragmatic motion:* the respiratory cycle is shown as a sinusoidal waveform. Diaphragmatic excursion (DE) was measured as the vertical distance between the end-inspiratory peak and the end-expiratory trough, indicated by the solid line.

DE was measured using M-mode ultrasound by a sonographer blinded to group allocation. With the patient in the supine position, a convex array probe (2–5 MHz) connected to an ultrasound system (Model A97H-03057, Konica Minolta Medical Printing Equipment (Shanghai) Co., Ltd., Shanghai, China) was placed below the right costal margin, using the liver as an acoustic window to visualize the diaphragm. Special care was taken to align the M-mode sampling line perpendicular to the motion of the posterior third of the hemidiaphragm to minimize angle-dependent measurement errors. M-mode curves were recorded during quiet breathing. DE was defined as the vertical distance between the peak at end-inspiration and the trough at end-expiration (26, 27) (Figure 3). Measurements were taken at three time points: pre-intervention (T0), during intervention (15–18 min, T1), and within 5 min post-intervention (T2). The average value of three respiratory cycles was calculated for each time point.

### Functional near-infrared spectroscopy (fNIRS) data acquisition

**Figure 4:**
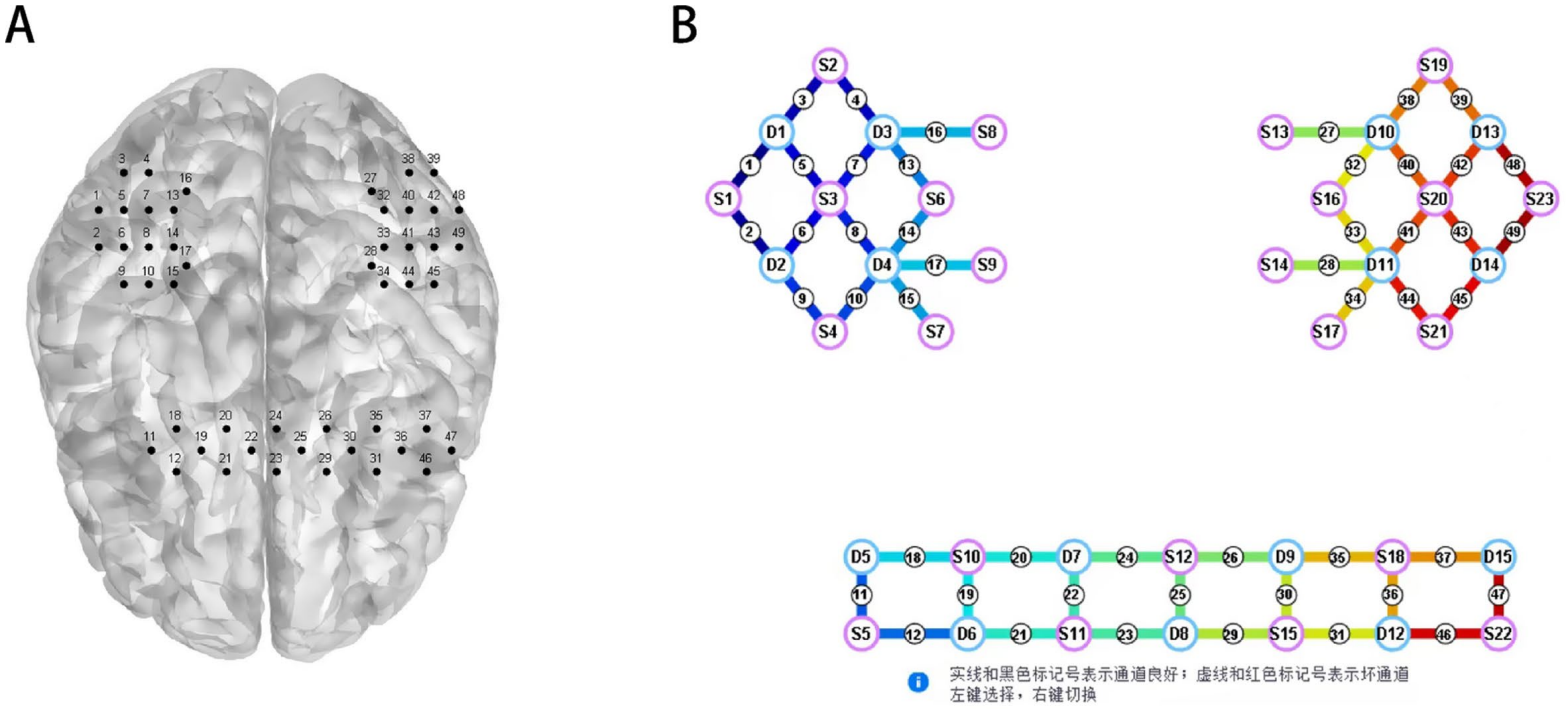
Configuration of fNIRS optodes and spatial registration. **(A)** 3D cortical projection: The spatial distribution of measurement channels is mapped onto a standard brain template, covering prefrontal and sensorimotor areas. **(B)** 2D topographic layout: Arrangement of sources (red circles), detectors (blue circles), and channels (numbered lines). Channels were grouped into five regions of interest (ROIs) according to the International 10–20 system: Dorsolateral prefrontal cortex (DLPFC), premotor cortex (PMC), primary motor cortex (M1), primary somatosensory cortex (S1), and somatosensory association cortex (SAC). The source-detector distance was set at 3.0 cm. The slight visual asymmetry in the optode arrangement is an artifact of the 2D projection of the 3D head model. The spatial registration was mapped onto the standard MNI152 brain template.

A multichannel fNIRS system (NirSmartll-6000A, Danyang Huichuang Medical Equipment Co., Ltd., Jiangsu, China) was used to record changes in the oxy-hemoglobin [HbO]. The system consists of a near-infrared light source (light-emitting diodes) and avalanche photodiodes as detectors. In this study, 23 light sources and 15 detectors comprised 49 active observation channels covering the cortical region of interest (ROI). Cortical activity was recorded using dual wavelength (730 and 850 nm) with a sampling rate of 11 Hz. The light sources and detectors were fixed using acquisition head caps, and the distance between them was set at an average distance of 3.0 cm. (Figure 4). Prior to the formal recording, a calibration procedure was performed to verify the optode-scalp coupling quality, and gain adjustments were made to ensure that light intensity for all channels met the device’s signal-to-noise ratio criteria. The study employed a block design paradigm. The fNIRS acquisition was synchronized with the EDNS intervention, consisting of three consecutive phases: (1) a 5-min pre-intervention resting-state baseline (T0); (2) a continuous 20-min EDNS intervention block (T1); and (3) a 5-min post-stimulation resting state (T2).

fNIRS data were processed offline using NirSpark software (v1.8.1). The comprehensive preprocessing pipeline included: (1) Data quality check and optical density conversion: Raw light intensity data were first converted into optical density (OD) changes. Channels with poor signal quality (e.g., excessively low signal-to-noise ratio or lack of clear cardiac pulsation) were identified and rejected. (2) Motion artifact correction: Spline interpolation was applied to detect and correct motion artifacts (parameters: amplitude threshold = 0.5, moving standard deviation = 6.0) (28). (3) Filtering: A 0.01–0.2 Hz band-pass filter was applied to remove physiological noise, including high-frequency cardiac oscillations and low-frequency respiratory drifts (19). (4) Hemodynamic calculation: Changes in [HbO] concentrations were calculated from the filtered OD data using the modified Beer–Lambert law (MBLL), with the differential pathlength factor (DPF) set to 6 (29). (5) Data segmentation: Task-related time windows were extracted, including a 5-min baseline, 20-min intervention, and 5-min post-intervention phase. The mean [HbO] signal from the 5-min pre-intervention resting state was defined as the zero-baseline for each channel to calculate the relative concentration changes during and after the intervention. It is worth noting that while standard General Linear Model (GLM) and block-averaging are conventionally used for event-related or short block-design fNIRS studies, our experimental paradigm involved a continuous 20-min neuromodulation intervention. For such prolonged and continuous stimulation, canonical hemodynamic response functions are often sub-optimal for modeling sustained state shifts. Therefore, consistent with established methodologies for long-duration continuous neuromodulation (such as tDCS or prolonged electrical stimulation), we utilized the mean relative changes in *Δ*[HbO] over the predefined continuous time segments to quantify sustained cortical activation (30, 31). (6) Activation analysis (Block Averaging): Rather than using a GLM which is optimized for short, alternating event/block designs via canonical hemodynamic response function (HRF) convolution, we employed a block averaging approach tailored for prolonged continuous interventions. The mean [HbO] concentrations were averaged across the predefined temporal blocks (5-min baseline, 20-min intervention, 5-min post-intervention). The relative concentration change (*Δ*[HbO]) was calculated by subtracting the averaged baseline from the intervention blocks. This block averaging metric is widely accepted for evaluating sustained cortical state changes induced by prolonged continuous neuromodulation, where standard HRF models may not accurately capture steady-state hemodynamic plateaus (32–34). (7) Spatial normalization for lesions: To eliminate the effect of lesion laterality, fNIRS channel coordinates for patients with right-sided lesions were horizontally mirrored along the mid-sagittal plane (35, 36). Consequently, in subsequent group-level analyses, “left” was defined as the ipsilesional side, and “right” as the contralesional side for all data.

The fNIRS optode cap was positioned strictly according to the international 10–20 system. Standard anatomical landmarks (e.g., nasion, inion, Cz, and preauricular points) were meticulously measured for each participant to ensure consistent cap placement. The correspondence between fNIRS channels and cortical Regions of Interest (ROIs) was established using a probabilistic spatial registration approach built into the NirSpark software, which maps the 10–20 system coordinates to standard Montreal Neurological Institute 152 (MNI152) brain template and their corresponding Brodmann Areas (BAs). Based on standard Brodmann areas, 10 ROIs were defined in this study: bilateral Primary Somatosensory Cortex (S1), Primary Motor Cortex (M1), Dorsolateral Prefrontal Cortex (DLPFC), Somatosensory Association Cortex (SAC), and Premotor Cortex (PMC) (ROI channel definitions are detailed in Table 1).

**Table 1:** Channel definitions for regions of interest (ROIs).

Region of interest (ROI)	Abbreviation	Channel numbers	Brodmann area (BA)	Count (*n*)
Right dorsolateral prefrontal cortex	R-DLPFC	9, 10, 19, 20	9,46	4
Left dorsolateral prefrontal cortex	L-DLPFC	35, 36, 44, 45	9,46	4
Right somatosensory association cortex	R-SAC	4	5,7	1
Left somatosensory association cortex	L-SAC	38	5,7	1
Right primary somatosensory cortex	R-S1	5, 7, 16	1,2,3	3
Left primary somatosensory cortex	L-S1	27, 40, 42	1,2,3	3
Right primary motor cortex	R-M1	13	4	1
Left primary motor cortex	L-M1	32	4	1
Right premotor cortex	R-PMC	6, 8, 14, 17	6	4
Left premotor cortex	L-PMC	28, 33, 41, 43	6	4

Probe placement follows the international 10–20 system. R, right hemisphere; L, left hemisphere.

### Statistical analysis

All statistical analyses were performed using SPSS 27.0 software (IBM Corp., Armonk, NY, USA). The normality of data distribution was rigorously assessed using the Shapiro–Wilk test. Continuous variables conforming to a normal distribution (e.g., Diaphragm Excursion) were expressed as mean ± standard deviation (SD), whereas non-normally distributed variables (e.g., fNIRS *Δ*[HbO] concentrations and clinical scores) were expressed as median [interquartile range, IQR]. Categorical variables were presented as counts (percentages). Baseline characteristics were compared using independent samples t-tests, Mann–Whitney U tests, or Chi-square tests, as appropriate.

A stratified statistical approach was applied based on data distribution. For normally distributed data, a 2 (Group) × 3 (Time) mixed-design Analysis of Variance (ANOVA) was conducted to evaluate main effects and interactions. The Greenhouse–Geisser correction was applied when the assumption of sphericity was violated. Post-hoc pairwise comparisons were performed using the Bonferroni correction to strictly control for multiple comparisons. For non-normally distributed data, the Friedman test was employed to evaluate longitudinal changes across time points within each group, and the Kruskal-Wallis H test was used for between-group comparisons. For post-hoc analysis, pairwise comparisons were conducted using the Wilcoxon signed-rank test (within-group) or Mann–Whitney U test (between-group) with a Bonferroni-adjusted significance threshold.

Correction for Spatial Multiple Comparisons (fNIRS): Given the high dimensionality of neuroimaging data, to rigorously control for the false positive rate across the 10 Regions of Interest (ROIs), the False Discovery Rate (FDR, Benjamini-Hochberg procedure) was applied to adjust the *p*-values for all spatial comparisons in the fNIRS analysis. Correlation analyses were performed using Spearman’s rank correlation coefficient to assess the relationships between cortical activation (*Δ*[HbO]) and clinical scores, as these datasets did not satisfy the assumption of normality. All tests were two-tailed, and a corrected *p*-value < 0.05 was considered statistically significant.

## Results

### Baseline characteristics of participants

A total of 50 stroke patients and 15 HC were enrolled in the study. Among the stroke patients, 30 (60%) had IS and 20 (40%) had ICH. Statistical analysis revealed no significant differences in age and sex distribution between the HC group and the combined stroke group (*p* > 0.05), indicating that the controls were well-matched. As shown in Table 2, comparison between stroke subtypes demonstrated that the IS and ICH groups were balanced regarding baseline clinical characteristics, including age, sex, hemiplegic side, Activities of Daily Living (ADL) scores, and Body Mass Index (BMI) (all *p* > 0.05).

**Table 2:** Baseline demographic and clinical characteristics of participants.

Characteristic	IS group (*n* = 30)	ICH group (*n* = 20)	HC group (*n* = 15)	*p* value
Age (years)	60.9 ± 11.6	54.5 ± 12.1	54.5 ± 10.6	0.09
Sex (Male), *n* (%)	19 (63%)	15 (75%)	10 (67%)	0.69
BMI (kg/m^2^)	22.7 ± 2.4	22.8 ± 2.7	21.8 ± 1.2	0.34
Hemiplegic side (Right), *n* (%)	16 (53.3%)	9 (45%)	NA	0.56
ADL score	41.6 ± 13.1	44.3 ± 14.3	NA	0.66

Data are presented as Mean ± Standard deviation (SD) or Number (Percentage). IS, ischemic stroke; ICH, intracerebral hemorrhage; HC, healthy controls; BMI, body mass index; ADL, activities of daily living (assessed by Barthel Index); NA, not applicable. Comparisons were performed using ANOVA, Chi-square test, or independent *t*-tests as appropriate.

### Changes in diaphragm excursion (DE)

A single session of EDNS significantly improved diaphragmatic activity in both patient groups, with no significant between-group difference in the temporal pattern of improvement. The mixed-design ANOVA revealed a significant main effect of Time (*p* < 0.01), indicating that the EDNS intervention effectively increased DE. However, the Group × Time interaction effect was not significant (*p* > 0.05), suggesting similar response trajectories between groups.

**Figure 5:**
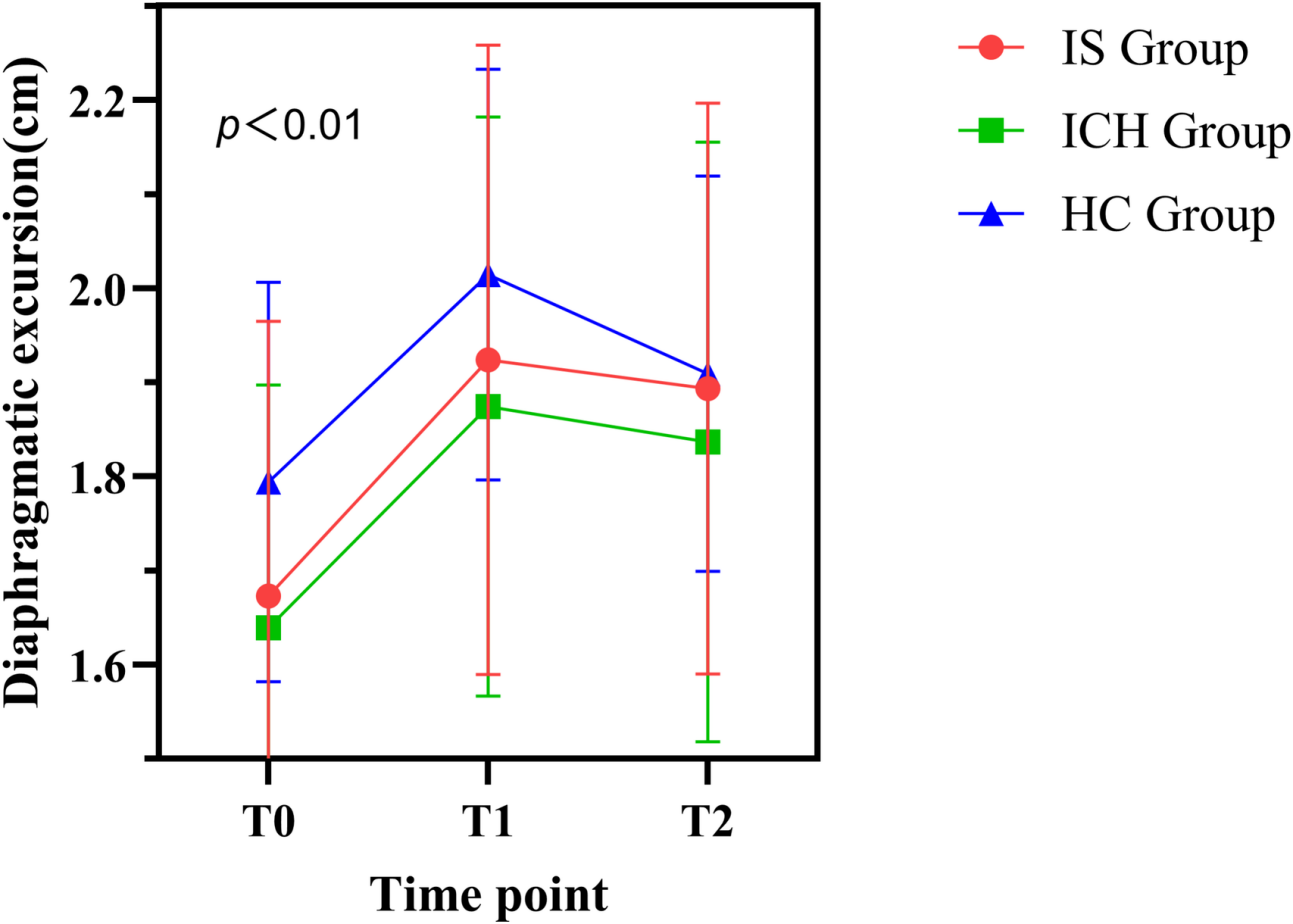
Temporal dynamic changes in diaphragm excursion (DE) across groups. The line graph displays the mean DE values for the Ischemic Stroke (IS, red circles), Intracerebral Hemorrhage (ICH, green squares), and Healthy Control (HC, blue triangles) groups at baseline (T0), during intervention (T1), and post-intervention (T2). Error bars represent standard deviation (SD). All groups showed a significant increase in DE during stimulation (T1) compared to baseline (*p* < 0.01).

Further within-group analysis (Table 3) showed that, compared with baseline (T0) (IS: 1.67 ± 0.29 cm; ICH: 1.64 ± 0.26 cm), DE significantly increased during stimulation (T1) (IS: 1.92 ± 0.33 cm; ICH: 1.87 ± 0.31 cm) and post-stimulation (T2) (IS: 1.89 ± 0.30 cm; ICH: 1.84 ± 0.32 cm) in both groups (*p* < 0.01, Figure 5).

**Table 3:** Changes in diaphragm excursion (DE) across time points.

	*n*	Baseline (T0)	During (T1)	After (T2)	Δ (T1- T0)	*p* value	Δ (T2- T0)	*p* value
IS group	30	1.67 ± 0.29	1.92 ± 0.33	1.89 ± 0.30	0.25 ± 0.17	<0.01	0.22 ± 0.21	<0.01
ICH group	20	1.64 ± 0.26	1.87 ± 0.31	1.84 ± 0.32	0.24 ± 0.13	<0.01	0.20 ± 0.14	<0.01
HC group	15	1.79 ± 0.21	2.01 ± 0.22	1.91 ± 0.21	0.22 ± 0.11	<0.01	0.12 ± 0.10	<0.01

Data are presented as Mean ± SD (unit: cm). T0, pre-intervention baseline; T1, during intervention (15–18 min); T2, immediately post-intervention. Delta, change from baseline. *p* values indicate statistical significance compared to within-group baseline (T0) using paired sample *t*-tests.

### Cortical activation patterns

Central cortical activation exhibited significant stroke subtype specificity: the IS group showed widespread activation, whereas the ICH group showed localized activation. Mixed-design ANOVA for [HbO] signals in the DLPFC and SAC showed no significant Group times Time interaction effects (*p* > 0.05), indicating that the dynamic patterns of central activation signals over time were similar between the two groups.

**Figure 6:**
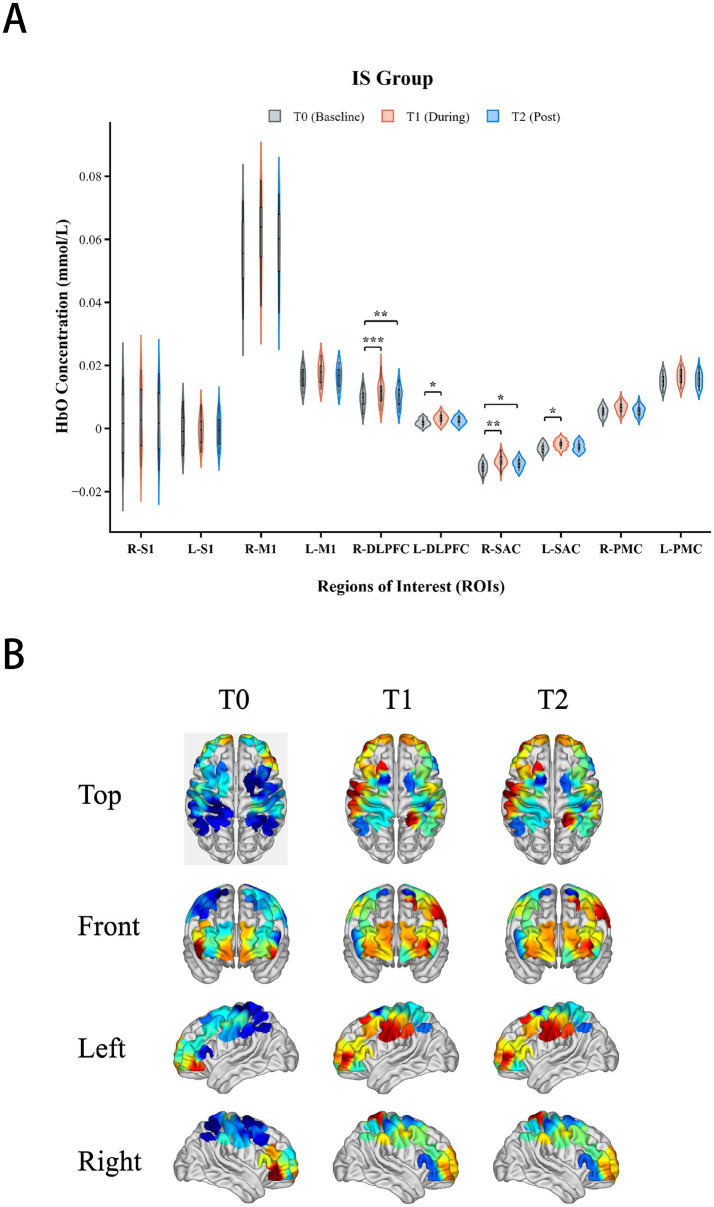
Cortical activation in the ischemic stroke (IS) group. **(A)** Violin plots showing the distribution of mean oxy-hemoglobin concentration differences (*Δ*[HbO]) across ROIs at different time points. **(B)** Corresponding topographic maps visualizing widespread activation in the bilateral dorsolateral prefrontal cortex (DLPFC) and bilateral somatosensory association cortex (SAC). *p < 0.05; ** *p* < 0.01; *** *p* < 0.001. Asterisks indicate significant differences compared to baseline (T0) (FDR corrected).

**Figure 7:**
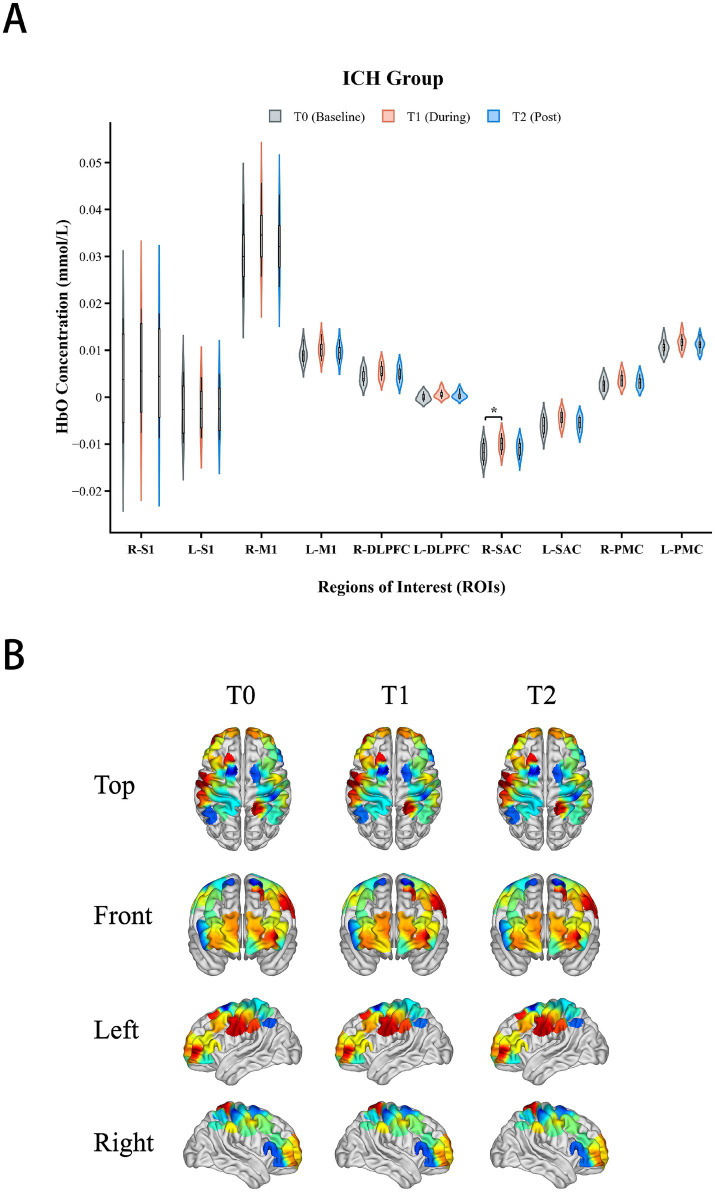
Cortical activation in the intracerebral hemorrhage (ICH) group. **(A)** Violin plots of mean (Δ[HbO]) differences across ROIs. **(B)** Corresponding topographic maps showing focal activation restricted to the right somatosensory association cortex (R-SAC), with no significant activation in prefrontal regions. *p < 0.05, **p < 0.01, ***p < 0.001. Asterisks indicate significant differences compared to baseline (T0) (FDR corrected).

However, the topology of activation (i.e., which brain regions were recruited) displayed significant heterogeneity. Within-group analysis (Table 4) revealed that the IS group exhibited significant activation in the bilateral DLPFC (Right: *p* = 0.024; Left: *p* = 0.048) and bilateral SAC (Right: *p* = 0.004; Left: *p* = 0.003) (Figure 6). In contrast, the ICH group showed significant activation only in the Right SAC (*p* = 0.046), with no significant activation observed in other ROIs (*p* > 0.05) (Figure 7).

**Table 4:** Changes in oxy-hemoglobin concentration [HbO] across all ROIs.

ROI	T0 (Baseline)	T1 (During)	T2 (After)	*p* value
Panel A: IS (*n* = 30)				
R-DLPFC M (Q1, Q3)	0.90 (−12.32, 9.21)	2.37 (−12.98, 9.77)	1.75 (−13.37, 10.08)	*0.024
L-DLPFCM (Q1, Q2)	−1.31 (−11.95, 11.49)	−1.18 (−10.09, 11.45)	−1.31 (−10.56, 12.23)	*0.048
R-SAC M (Q1, Q3)	−7.67 (−51.65, 10.40)	−6.65 (−52.78, 10.89)	−7.09 (−51.01, 11.93)	*0.004
L-SAC M (Q1, Q3)	−4.95 (−19.27, 7.91)	−3.19 (−19.80, 7.70)	−3.95 (−19.43, 6.77)	*0.003
R-S1 M (Q1, Q3)	−4.70 (−16.69, 8.83)	−3.69 (−17.55, 9.17)	−4.70 (−17.41, 9.89)	0.875
L-S1 M (Q1, Q3)	−1.55 (−18.37, 9.91)	−1.24 (−18.55, 9.90)	−1.34 (−18.18, 10.29)	0.996
R-M1 M (Q1, Q3)	0.99 (−21.66, 24.39)	1.07 (−21.71, 26.84)	0.42 (−22.88, 27.36)	0.195
L-M1 M (Q1, Q3)	−2.76 (−30.87, 12.61)	−3.14 (−30.50, 13.40)	−3.44 (−31.30, 13.53)	0.393
R-PMC M (Q1, Q3)	−4.55 (−21.44, 4.00)	−4.98 (−21.49, 4.85)	−4.67 (−21.68, 4.35)	0.792
L-PMC M (Q1, Q3)	−1.45 (−9.54, 11.85)	−0.93 (−7.96, 10.96)	−0.67 (−8.23, 11.43)	0.321
Panel B: ICH (*n* = 20)				
R-DLPFC M (Q1, Q3)	5.85 (−3.96, 13.78)	6.16 (−3.80, 14.98)	5.61 (−3.48, 15.10)	0.287
L-DLPFC M (Q1, Q2)	4.04 (−5.70, 28.07)	4.30 (−5.29, 28.15)	3.83 (−4.57, 28.73)	0.835
R-SAC M (Q1, Q3)	−11.60 (−33.58, 13.65)	−10.26 (−34.9, 12.71)	−10.80 (−34.54, 12.77)	*0.046
L-SAC M (Q1, Q3)	−10.13 (−37.22, 23.58)	−9.56 (−38.04, 22.92)	−4.45 (−37.41, 23.26)	0.705
R-S1 M (Q1, Q3)	0.64 (−24.66, 12.56)	0.62 (−21.79, 12.73)	1.10 (−20.33, 12.75)	0.163
L-S1 M (Q1, Q3)	3.36 (−7.59, 20.85)	4.28 (−7.37, 20.92)	4.09 (−6.94, 20.75)	0.165
R-M1 M (Q1, Q3)	−5.28 (−22.72, 15.37)	−2.29 (−23.95, 15.77)	−6.60 (−23.67, 16.80)	0.549
L-M1 M (Q1, Q3)	−3.90 (−19.91, 22.66)	−2.50 (−19.19, 22.76)	−3.30 (−20.02, 20.69)	0.96
R-PMC M (Q1, Q3)	3.03 (−8.87, 16.69)	2.94 (−8.99, 17.27)	3.27 (−9.86, 15.88)	0.413
L-PMC M (Q1, Q3)	−1.74 (−13.84, 8.27)	−0.47 (−14.37, 8.52)	−1.12 (−15.03, 8.82)	0.165
Panel C: HC (*n* = 15)				
R-DLPFC M (Q1, Q3)	−22.25 (−68.69, 11.83)	−24.89 (−69.39, 11.79)	−24.66 (−70.31, 11.96)	0.842
L-DLPFC M (Q1, Q2)	−4.22 (−42.92, 8.60)	−3.17 (−41.57, 7.18)	−3.43 (−40.36, 6.70)	0.672
R-SAC M (Q1, Q3)	17.93 (−25.57, 99.79)	16.63 (−24.55, 102.16)	16.30 (−24.45, 109.69)	0.694
L-SAC M (Q1, Q3)	27.72 (−70.03, 60.72)	27.86 (−69.27, 62.97)	20.66 (−71.80, 62.36)	0.552
R-S1 M (Q1, Q3)	−12.68 (−63.15, 33.60)	−12.07 (−63.90, 35.16)	−13.54 (−62.17, 35.64)	0.299
L-S1 M (Q1, Q3)	−3.75 (23.03, 49.45)	−2.55 (21.19, 55.03)	−3.74 (−21.53, 55.08)	0.378
R-M1 M (Q1, Q2)	−9.30 (−76.61, 31.96)	−8.49 (−74.46, 33.49)	−7.98 (−77.91, 34.07)	0.655
L-M1 M (Q1, Q3)	7.73 (−85.38, 67.93)	7.88 (−88.48, 68.00)	6.09 (−88.47, 69.89)	0.420
R-PMC M (Q1, Q3)	0.16 (−27.49, 12.31)	−0.62 (−26.45, 12.30)	−0.16 (−30.25, 11.64)	0.575
L-PMC M (Q1, Q3)	2.78 (−23.77, 20.27)	2.67 (−21.36, 21.30)	1.58 (−22.46, 20.20)	0.099

Data are presented as Median [Interquartile Range (Q1, Q3)]. Unit for [HbO] concentration change is 10–3 mmol/L. Abbreviations: R: Right; L: Left; DLPFC: Dorsolateral Prefrontal Cortex; SAC: Somatosensory Association Cortex; M1: Primary Motor Cortex; S1: Primary Somatosensory Cortex; PMC: Premotor Cortex. Time points: T0: Baseline; T1: During EDNS; T2: After EDNS. *p < 0.05; **p < 0.01 (*P* values for within-group longitudinal changes were derived from the Friedman test. FDR corrected for multiple comparisons across 10 ROIs).

### Central-peripheral coupling

To investigate the functional significance of the differences in central activation topology, we further analyzed the central-peripheral correlation. In the IS group, the magnitude of DE improvement (T1-T0) was positively correlated with the activation intensity of the Right DLPFC (R-DLPFC) (*ρ* = 0.386, *p* = 0.035). Conversely, in the ICH group, no significant correlation was found between the activation of the Right SAC (R-SAC) and the magnitude of DE improvement (*ρ* = 0.329, *p* = 0.157; Table 5).

**Table 5:** Correlation between diaphragm excursion improvement and cortical activation intensity.

Group	ROI	*n*	*ρ* (Spearman)	*p* value
IS group	R-DLPFC	30	0.386	*0.035
IS group	L-DLPFC	30	0.050	0.792
IS group	R-SAC	30	0.080	0.965
IS group	L-SAC	30	0.126	0.505
ICH group	R-SAC	20	0.329	0.157

Analysis was performed using Spearman’s rank correlation coefficient (*ρ*). Variables: Change in Diaphragm Excursion (DE) and change in oxy-hemoglobin [HbO] concentration were calculated as the difference between the stimulation period (T1) and baseline (T0) (Delta = T1 - T0). * Indicates statistical significance (*p* < 0.05).

### Diaphragmatic and cortical responses in healthy controls

Regarding peripheral effects, the HC group demonstrated significant improvement in diaphragmatic function. Compared with the pre-stimulation baseline (1.79 ± 0.21 cm), DE significantly increased during EDNS stimulation (2.01 ± 0.22 cm) and post-stimulation (1.91 ± 0.21 cm) (*p* = 0.001).

**Figure 8:**
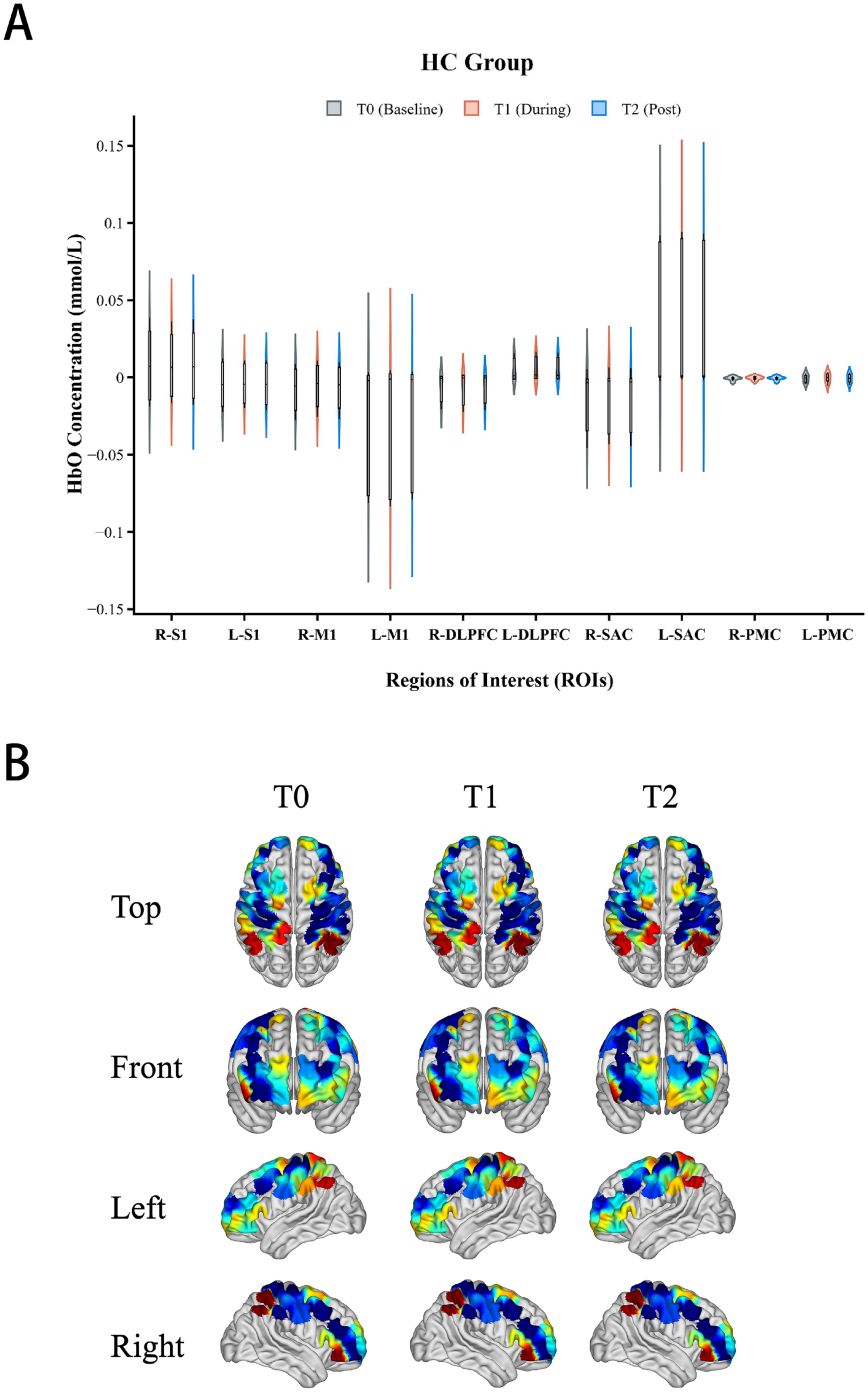
Cortical activation in the healthy control (HC) group. **(A)** Violin plots of mean (Δ[HbO]) differences indicating no significant changes compared to baseline. **(B)** Corresponding topographic maps displaying a “silent” cortical response with no distinct activation clusters.

Repeated measures ANOVA showed no significant changes in [HbO] concentration in any of the monitored ROIs during or after EDNS intervention (*p* > 0.05, Figure 8).

## Discussion

This study investigated the immediate central-peripheral effects of EDNS on different stroke subtypes (IS vs. ICH) using simultaneous fNIRS and ultrasound monitoring. The core finding is that the effects of EDNS manifest as a form of “pattern separation”: while the peripheral function and central temporal patterns were consistent between groups, the topology of central activation and the central-peripheral coupling differed significantly.

The first key finding of this study is the universality of EDNS in improving peripheral diaphragmatic function. The data showed that a single session of EDNS immediately and significantly increased DE in both IS and ICH patients (main effect of Time, *p* < 0.01). Notably, the “Group × Time” interaction was not significant (*p* > 0.05; Table 6). This result objectively reflects the high homogeneity in the temporal dynamics of diaphragmatic improvement between the two stroke subtypes, suggesting that despite significant differences in central pathological mechanisms, the peripheral diaphragmatic response to EDNS remains consistent. This physiological response, independent of specific central pathologies, suggests that EDNS has good clinical generalizability as a fundamental respiratory rehabilitation technique. The mechanism likely lies in EDNS being a peripheral intervention that primarily relies on the intact phrenic nerve-diaphragm pathway, allowing stimulation to bypass damaged corticospinal tracts and directly drive the diaphragm (37, 38).

**Table 6:** Analysis of temporal dynamics and interaction effects (IS vs. ICH).

Variable	Time point	IS group (*n* = 30)	ICH group (*n* = 20)	Interaction *P* value
A. Cortical Activation [HbO]		M (Q1, Q3)	M (Q1, Q3)	(Group × Time)
R-DLPFC	T0	0.90 (−12.32, 9.21)	5.85 (−3.96, 13.78)	> 0.05
	T1	2.37 (−12.98, 9.77)	6.16 (−3.80, 14.98)	
	T2	1.75 (−13.37, 10.08)	5.61 (−3.48, 15.10)	
L-DLPFC	T0	−1.31 (−11.95, 11.49)	4.04 (−5.70, 28.07)	> 0.05
	T1	−1.18 (−10.09, 11.45)	4.30 (−5.29, 28.15)	
	T2	−1.31 (−10.56, 12.23)	3.83 (−4.57, 28.73)	
R-SAC	T0	−7.67 (−51.65, 10.40)	−11.60 (−33.58, 13.65)	> 0.05
	T1	−6.65 (−52.78, 10.89)	−10.26 (−34.91, 12.71)	
	T2	−7.09 (−51.01, 11.93)	−10.80 (−34.54, 12.77)	
L-SAC	T0	−4.95 (−19.27, 7.91)	−10.13 (−37.22, 23.58)	> 0.05
	T1	−3.19 (−19.80, 7.70)	−9.56 (−38.04, 22.92)	
	T2	−3.95 (−19.43, 6.77)	−4.45 (−37.41, 23.26)	
B. Diaphragm function (DE)		Mean ± SD	Mean ± SD	(Group × Time)
Diaphragm excursion (cm)	T0	1.67 ± 0.29	1.64 ± 0.26	> 0.05
	T1	1.92 ± 0.33	1.87 ± 0.31	
	T2	1.89 ± 0.30	1.84 ± 0.32	

Data Presentation: Cortical activation data (Panel A) are non-normally distributed and presented as Median (Q1, Q3). Diaphragm data (Panel B) are normally distributed and presented as Mean ± SD. Units: [HbO] concentration in 10^−3^ mmol/L; Diaphragm Excursion (DE) in cm. Statistics: The interaction *P* value assesses whether the trend of change over time differs between groups. *p* > 0.05 indicates no significant difference in the dynamic response pattern between the IS and ICH groups. The interaction *p* values were derived from the mixed-design ANOVA.

The specificity of this central mechanism was further confirmed by the inclusion of the healthy control (HC) group. A critical finding is that, compared with the stroke groups, the cortex of healthy controls appeared relatively “silent” under EDNS intervention. This phenomenon likely reflects the efficiency or automaticity of the healthy nervous system in processing such sensory inputs (39–41). With intact neural pathways, phrenic nerve afferents may be processed primarily through brainstem-spinal loops or subcortical structures without recruiting higher-order cortical resources like the DLPFC. While the relatively small sample size of the HC group (*n* = 15) warrants cautious interpretation regarding potential Type II errors, this observed ‘cortical silence’ stands in sharp contrast to the widespread activation seen in the IS group. This inversely corroborates that the DLPFC-SAC cortical activation patterns activation observed in the IS group is not a physical artifact or routine physiological response, but rather a compensatory neural recruitment in a pathological state (21, 42). Specifically, when ischemia compromises the efficiency of original pathways, the brain is compelled to recruit the DLPFC, a region typically responsible for higher-order cognitive control, to assist in processing sensory inputs. In contrast, the ICH group lacked both the efficient automaticity of healthy individuals and the concurrent cortical compensatory activation seen in the IS group, resulting in a “disconnection” of central-peripheral regulation. This comparison across HC, IS, and ICH groups clearly delineates a neural regulatory spectrum ranging from “physiological efficiency” to “pathological compensation” and finally to “cortical uncoupling.”

The most critical finding of this study lies in the stroke subtype specificity of the central effects of EDNS. Although the temporal patterns of central activation were consistent (interaction *p* > 0.05), the spatial topology was distinctly different. IS patients exhibited activation of “cognitive-sensory” cortical regions involving the bilateral DLPFC and SAC. The DLPFC is associated with higher cognitive functions such as attention and working memory, and its involvement in sensorimotor tasks reflects the processing of afferent information by the cognitive control Cortex (43). Previous studies have shown that afferent sensory stimulation (e.g., phrenic nerve stimulation) can induce plastic reorganization in motor and prefrontal regions (44, 45). Therefore, the synergistic DLPFC-SAC activation observed in the IS group likely reflects not merely simple sensory processing, but the Cerebral cortex for rapid plastic reorganization in response to peripheral input. Conversely, the activation pattern in ICH patients was extremely localized, limited to the right SAC (*p* = 0.046). This topological heterogeneity may stem from the unique pathophysiology of ICH. Compared with focal ischemia, intracerebral hemorrhage often causes more extensive damage to subcortical white matter tracts and diffuse neurotoxicity, which may exert stronger functional inhibition on higher-order association cortices like the DLPFC that require high connectivity, rendering them difficult to recruit by EDNS intervention (21, 46). Thus, the central response in IS patients presents as “cognitive-sensory” coupling, whereas the response in ICH patients is limited to basic sensory processing.

Beyond the higher-order association cortices, we also closely examined the sensorimotor execution cortical activation patterns. Although no statistically significant group-level activation was observed in the primary motor cortices (M1) of the ICH group (*p* > 0.05), a notable discrepancy in signal variance was visually evident between the contralesional (Right) and ipsilesional (Left) M1. Because all lesions were spatially normalized to the left hemisphere, L-M1 represents the ipsilesional cortex. Its relatively uniform and suppressed hemodynamic response is likely attributable to direct structural disruption or severe diaschisis common in ICH. Conversely, the contralesional R-M1 exhibited substantial inter-subject variability (large signal variance). This large discrepancy likely reflects the heterogeneous nature of post-stroke compensatory mechanisms. Disruption of the affected hemisphere can lead to varying degrees of reduced interhemispheric inhibition, causing hyper-excitability or highly variable compensatory activation in the contralesional M1 across different patients. However, since these signal fluctuations in R-M1 did not reach statistical significance at the group level, it further corroborates our conclusion that EDNS primarily drives basic sensory processing in ICH, rather than consistently recruiting the motor execution network.

Differences in central topology are also reflected in the heterogeneity of central-peripheral coupling. We found that in the IS group, the intensity of Right DLPFC activation was positively correlated with the magnitude of DE improvement (*ρ* = 0.386, *p* = 0.035). This suggests that IS patients may preserve a “top-down” cognitive regulatory mechanism, where DLPFC activation participates in modulating the peripheral diaphragmatic response. In contrast, in the ICH group, the solely activated R-SAC showed no significant correlation with DE improvement (*p* = 0.157). This central-peripheral “dissociation,” combined with the absence of DLPFC involvement, may reflect a non-linear threshold regulation mode: SAC activation serves merely as a basic step in processing afferent stimuli rather than playing the linear regulatory role of the DLPFC. This finding reveals two potential neural regulatory pathways: IS patients retain a cognitive regulatory pathway, while the response in ICH patients tends toward basic sensory input processing.

However, it is crucial to acknowledge the domain-general nature of the DLPFC. As a core hub of the frontoparietal control network, the DLPFC is heavily recruited across a myriad of experimental paradigms involving attention, executive function, and increased cognitive load. Consequently, its isolated activation lacks the high specificity required to serve as a definitive, standalone clinical biomarker for EDNS efficacy. In the context of our study, we do not posit the DLPFC as a highly specific diagnostic target, but rather interpret its widespread activation as a neurophysiological signature reflecting the increased top-down cognitive resources and attentional effort required by IS patients to process novel peripheral sensory inputs following the disruption of lower-level sensorimotor Cortex. To achieve true clinical utility and support decision-making, future studies must interpret this prefrontal activation in conjunction with modality-specific physiological metrics (e.g., diaphragm excursion) and longitudinal clinical behavioral assessments.

The results of this study have potential clinical implications. Given that EDNS activates stable cortical-diaphragmatic pathways across different pathologies, future research could explore its combination with task-oriented training or brain-computer interfaces (BCI) to promote synergistic respiratory-motor recovery.

Beyond guiding personalized rehabilitation strategies, our findings provide a potential neurophysiological reference for clinical evaluation. The specific concurrent DLPFC-SAC cortical activation pattern observed in IS patients not only reveals a compensatory regulatory mechanism, but its presence or intensity could serve as an observational metric for the central response to respiratory rehabilitation. For instance, IS patients who fail to exhibit concurrent activation in these cognitive-sensory regions under EDNS might have a central response pattern closer to the ICH group. Future longitudinal studies should explore whether this baseline fNIRS activation pattern can be used for patient stratification to screen for the population most likely to benefit from synergistic cognitive-respiratory training.

This study has several limitations. First, the relatively limited sample size may reduce statistical power. Second, the lack of a sham stimulation control group makes it difficult to completely rule out placebo effects or somatosensory confounds. Third, the limited penetration depth of fNIRS prevents the capture of activity in deep nuclei such as the brainstem respiratory centers (47). Fourth, due to the lack of individual 3D digitization of optode positions or subject-specific MRI structural coregistration, the spatial mapping of channels to cortical regions relied on a standard probabilistic template based on the 10–20 system. While this is a widely accepted approach in clinical fNIRS studies, it may not fully account for individual anatomical variations or post-stroke cortical deformation. Consequently, while we successfully mapped the cortical correlates of EDNS, the potential relay role of subcortical structures such as the thalamus or brainstem respiratory centers remains to be elucidated through multi-modal imaging approaches. Future studies should focus on larger randomized controlled trials and potentially combine fMRI to explore subcortical mechanisms and broader cortical activation patterns.

## Conclusion

In conclusion, this study found that a single session of EDNS immediately improves diaphragmatic function in both ischemic and intracerebral hemorrhage stroke patients, with consistent temporal dynamics in peripheral effects and central activation. However, the underlying central neuromodulatory mechanisms exhibit significant pathological specificity: ischemic stroke recruits a widespread “cognitive-sensory” compensatory cortical activation patterns (DLPFC-SAC) and preserves central-peripheral coupling, whereas hemorrhagic stroke induces only a localized sensory cortical response. This indicates that while EDNS universally drives peripheral respiratory muscles, the induced pattern of central reorganization varies by stroke subtype, highlighting the need to consider neural regulatory differences underlying different pathologies when formulating respiratory rehabilitation strategies.

## Data Availability

The original contributions presented in the study are included in the article/supplementary material, further inquiries can be directed to the corresponding author/s.
